# The complete chloroplast genome of *Hibiscus syriacus* using long-read sequencing: Comparative analysis to examine the evolution of the tribe Hibisceae

**DOI:** 10.3389/fpls.2023.1111968

**Published:** 2023-02-02

**Authors:** Hyunjin Koo, Ah-Young Shin, Seongmin Hong, Yong-Min Kim

**Affiliations:** ^1^ Plant Systems Engineering Research Center, Korea Research Institute of Bioscience and Biotechnology, Daejeon, Republic of Korea; ^2^ Department of Bioinformatics, Korea Research Institute of Bioscience and Biotechnology (KRIBB) School of Bioscience, Korea University of Science and Technology (UST), Daejeon, Republic of Korea; ^3^ Digital Biotech Innovation Center, Korea Research Institute of Bioscience and Biotechnology (KRIBB), Daejeon, Republic of Korea

**Keywords:** long-read sequencing platform, complete chloroplast genome assembly, *Hibiscus syriacus*, comparative analysis, Hibisceae

## Abstract

*Hibiscus syriacus*, a member of the tribe Hibisceae, is considered an important ornamental and medicinal plant in east Asian countries. Here, we sequenced and assembled the complete chloroplast genome of *H. syriacus* var. *Baekdansim* using the PacBio long-read sequencing platform. A quadripartite structure with 161,026 base pairs was obtained, consisting of a pair of inverted repeats (IRA and IRB) with 25,745 base pairs, separated by a large single-copy region of 89,705 base pairs and a short single-copy region of 19,831 base pairs. This chloroplast genome had 79 protein-coding genes, 30 transfer RNA genes, 4 ribosomal RNA genes, and 109 simple sequence repeat regions. Among them, *ndhD* and *rpoC1*, containing traces of RNA-editing events associated with adaptive evolution, were identified by analysis of putative RNA-editing sites. Codon usage analysis revealed a preference for A/U-terminated codons. Furthermore, the codon usage pattern had a clustering tendency similar to that of the phylogenetic analysis of the tribe Hibisceae. This study provides clues for understanding the relationships and refining the taxonomy of the tribe Hibisceae.

## 1 Introduction

*Hibiscus* is one of the most diverse and widespread genera in the Malvaceae tribe Hibisceae (Rizk and Soliman, 2014). The members of the tribe Hibisceae are widely distributed from tropical to temperate regions worldwide (Akpan, 2000). Several species of the tribe Hibisceae are regarded as valuable research crops since they are economically important for food and medicines and can be utilized as biofuels due to their high biomass content and photosynthetic efficiency (Akpan et al., 2000; Saba et al., 2015; Wu et al., 2017; Cheng et al., 2020). *Hibiscus syriacus*, a member of the tribe Hibisceae, is a flowering shrub that originated in the Korean peninsula and southern China. It is one of the most widely planted ornamental species in temperate zones and is a fast-growing species with attractive white, red, pink, purple, and lavender flowers (Paoletti et al., 2009; Kim et al., 2017). Along with ornamental value, the dried flowers and root bark of *H. syriacus* have been used as a traditional remedy in Oriental countries (Yoo et al., 1998). Particularly, three naphthalene chemicals (syriacusins A–C) and novel pentacyclic triterpene esters identified from the plant’s root bark have been used as anthelmintic, antipyretic, and antifungal agents (Yoo et al., 1998; Yun et al., 1999).

Chloroplasts are multifunctional organelles that carry their own genetic sources responsible for photosynthesis, various types of metabolism, and carbon fixation (Li et al., 2019). Chloroplast genomes typically have a quadripartite structure with two copies of inverted repeat (IR) regions separating the large and small single-copy (LSC and SSC, respectively) regions. Most chloroplast genomes range from 120 to 160 kb. Generally, chloroplast genomes of angiosperms contain approximately 120 genes including protein-coding genes, transfer RNA (tRNA), and ribosomal RNA (rRNA) (Daniell et al., 2016). Several mutational events, including mutations, duplications, rearrangements, and gene deletions, occur in chloroplast genomes (Lee et al., 2007). Nevertheless, compared to the nuclear or mitochondrial genome, the chloroplast genome is structurally conserved; hence, it is commonly employed to elucidate the genome evolution and phylogenetic relationships of land plants (Huang et al., 2014; Wu et al., 2017). With the emergence of high-throughput sequencing, the chloroplast genome assemblies of various species of the tribe Hibisceae have been completed (Cheng et al., 2020; Li et al., 2020; Mehmood et al., 2020). Although the phylogenetic relationship among several species of the family Malvaceae was estimated in previous studies, it is insufficient that a comprehensive comparative analysis of the chloroplast genomes in the tribe Hibisceae.

Here, we report the whole chloroplast genome of *H. syriacus* var. *Baekdansim* (hereafter referred to as Baekdansim) using PacBio long-read sequencing data for the first time. Further comparative genome analyses were carried out using the complete chloroplast genomes of other species belonging to the tribe Hibisceae that were obtained from the NCBI database. The findings of this study will be helpful for the development of genetic markers to resolve taxonomic discrepancies or to infer phylogenetic and evolutionary relationships within the tribe Hibisceae.

## 2 Materials and methods

### 2.1 Plant material and chloroplast DNA extraction

To isolate high-purity Baekdansim chloroplast DNA from cells, chloroplasts and mitochondria were the first separated from other components, especially nucleus DNA. This step was achieved by homogenizing 5–10 g (fresh weight) of young leaf tissue followed by a nuclei isolation step according to previous protocols (Zerpa-Catanho et al., 2021). For chloroplast DNA extraction, nuclei removed extract was transferred to 10 mL lysis buffer (50 mM Tris-HCl pH 7.5, 1.4 M NaCl, 20 mM EDTA, pH 8.0, 0.5% SDS) and incubated for 1 h in a 65°C water bath with gentle inversion every 20 min. The supernatant was separated by centrifugation at 3000 rpm for 10 min and transferred to a new tube. RNase A (10 mg/mL) was then added, and the mixture was incubated for 30 min at room temperature. Next, an equal volume of phenol:chloroform:isoamyl alcohol (25:24:1) was added to the supernatant, and the sample was mixed by gentle inversion for 5 min before centrifugation at 3000 rpm for 10 min. After the aqueous phase was transferred to a new tube, an equal volume of chloroform was added and mixed. The mixture was separated by centrifugation at 3000 rpm for 10 min. The upper, DNA-containing phase was transferred to a new tube, and an equal volume of isopropanol was added to precipitate the DNA, followed by centrifugation at 3000 rpm for 5 min. The DNA pellet was washed with 70% ethanol and resuspended in 100 µL of TE buffer (pH 8.0). Solubilized DNA was stored at 4°C until library preparation.

### 2.2 Library construction and sequencing

Purified genomic DNA (gDNA) was used for library construction with the SMRTbell Express Template Prep Kit (Pacific Biosciences, Cat. No. 101-357-000). In brief, gDNA was mechanically sheared to an average size of 20 kb using a Covaris g-TUBE device (Part No. 520079). In total, 5 μg of sheared gDNA was damage-repaired and end-repaired using polishing enzymes. Blunt-end adapter ligation was used to create the SMRTbell template. Adapter dimers and contaminants were removed using the AMPure XP bead purification system (Beckman Coulter, Cat. No. A63882). A BluePippin size selection system (Sage Science, Cat. No. BLU0001) was used to size select the SMRTbell template and enrich for fragments > 15 kb. Sequencing primer v4 was annealed to the SMRTbell template, and a DNA polymerase/template complex was created using the Sequel Binding Kit 2.1 (Pacific Biosciences, Cat. No. 101-365-900). An additional AMPure XP purification step was performed to remove excess primer and polymerase prior to sequencing. The library was sequenced on a Sequel instrument using SMRT Cell 1M v2 (Pacific Biosciences), taking one movie of 10 hours per cell with the Sequel Sequencing Kit 2.0 (Pacific Biosciences).

### 2.3 Genome assembly and annotation

**Figure 1:**
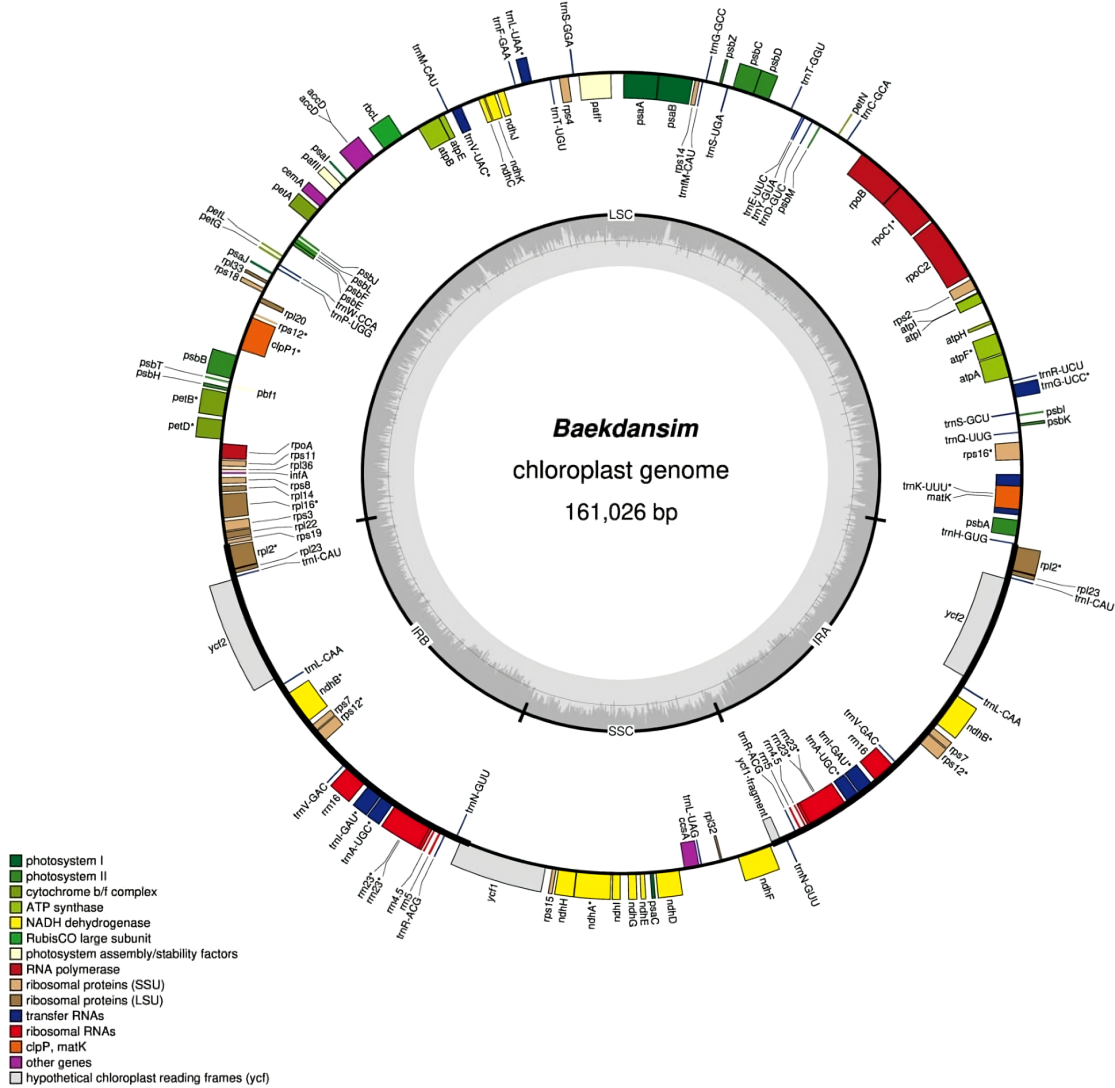
Circular chloroplast genome map of *H. syriacus* var. *Baekdansim*. The inner grey circle indicates the proportion of GC in each region. The genes illustrated in the inner circle are transcribed clockwise. Genes corresponding to distinct functional groups are denoted using distinct colors.

Reads from chloroplasts were extracted by alignment of all reads onto the five chloroplast complete genome assemblies of *Hibiscus* species (*H. syriacus*: NC_026909.1*, H. cannabinus*: NC_045873.1, *H. trionum*: NC_060636.1, *H. rosa-sinensis*: NC_042239.1, and *H. taiwanensis*: NC_045873.1) deposited in the NCBI database (https://www.ncbi.nlm.nih.gov/nucleotide/). Each chloroplast genome was duplicated and concatenated to facilitate the alignment of reads on the circularized region as suggested by Wang et al. (Wang et al., 2018). Long reads were mapped to chloroplast genomes using minimap2 version 2.24 (Li, 2018). Then, the short reads were mapped using bwa version 0.7.17 (Li and Durbin, 2009). A data set of extracted chloroplast reads was constructed using Unicycler v0.5.0 with the hybrid assembly strategy (Wick et al., 2017). Genome annotation was performed on the GeSeq platform using the complete chloroplast genome (Tillich et al., 2017). Coding sequence (CDS) and rRNAs were predicted by BLAT (Kent, 2002) and HMMER (Finn et al., 2011) search. In addition, the tRNAs were further verified by tRNAscan-SE v2.0.7 (Lowe and Chan, 2016) and ARAGORN v1.2.38 (Laslett and Canback, 2004) with default option. Then, a circular chloroplast map was constructed according to the genome annotation using the online program OGDRAW v1.3.1 (Greiner et al., 2019). The final Baekdansim plastome was deposited in GenBank with accession number OP874596.1. The corresponding circular genome map is shown in **Figure 1**.

### 2.4 Repeat sequence identification

Two programs were used to detect repeat motifs. Regarding microsatellites, MISA software (Beier et al., 2017) was used to examine the locations and motifs of simple sequence repeats (SSRs). SSRs were detected using thresholds of 10, 5, 4, 3, 3 and 3 repeat units for mono-, di-, tri-, tetra-, penta-, and hexa-nucleotides, respectively. To identify long repeat motifs, forward, reverse, complementary, and palindromic sequences were determined using REPuter v1.0, with a minimum repetition size of 30 bp and 90% identity (Kurtz et al., 2001).

### 2.5 Genetic divergence and chloroplast genome comparison

The nucleotide divergence (π) among the 13 species of the tribe Hibisceae was determined using DnaSP v6.0 based on sliding window analysis (**Supplementary Table 1**) (Rozas et al., 2017). The window length was set to 600 bp with a 100-bp step size. Comprehensive alignments of the complete chloroplast genomes of the tribe Hibisceae were examined using the mVISTA program (Frazer et al., 2004) to reveal interspecific variations. Furthermore, expansion and contraction between the LSC/IRB/SSC/IRA regions at junction sites were identified using IRscope (Amiryousefi et al., 2018). Genes in the chloroplast genomes of 13 species were investigated to determine the presence of introns. Alterations of genes containing intron regions were identified using in-house Python code.

### 2.6 Codon usage and RNA-editing sites

Relative synonymous codon usage (RSCU) analysis of coding sequences was conducted using MEGA v11.0 (Kumar et al., 1994), and an RSCU value greater than one was regarded as a high codon frequency. The putative RNA-editing sites of the start and stop codons of the coding sequences from species of the tribe Hibisceae were predicted using in-house Python code.

### 2.7 Phylogenetic analysis

The complete chloroplast genome sequence of Baekdansim, together with those of the other 12 species of the tribe Hibisceae available in the NCBI database, were used for comparative and phylogenetic analyses (**Supplementary Table 1**). The chloroplast sequence of *Gossypium hirsutum* (NC_007944) was also included as an outgroup (**Supplementary Table 1**). All chloroplast sequences were aligned in MAFFT using default parameters. The best-fit model (K3Pu+F+R4) was estimated using ModelFinder (Kalyaanamoorthy et al., 2017) with Bayesian Information Criterion (BIC) implemented in IQ-TREE v2.0.3 (Minh et al., 2020). Based on the best-fit model (K3Pu+F+R4), we inferred a maximum likelihood tree with 1,000 bootstrap replicates using IQ-TREE. The tree was rooted at midpoint and visualized using FigTree v.1.4.4 (http://tree.bio.ed.ac.uk/software/figtree/).

## 3 Results and discussion

### 3.1 Chloroplast genome assembly

The complete chloroplast genome of Baekdansim was constructed using the PacBio long-read sequencing platform (**Figure 1**). Due to the high-quality sequence data provided by the PacBio long-read sequencing technique and its capacity to assemble long reads, a single contig and, ultimately, the whole chloroplast genome of *H. syriacus* could be extracted (Chin et al., 2013). The complete chloroplast genome was 161,026 bp and had a quadripartite structure, including a pair of IR regions (IRA and IRB) separated by an LSC (89,705 bp) region and an SSC (19,831 bp) region. The SSC region in the genome assemblies of species of the family Malvaceae in the NCBI database, as determined using short reads, are usually bidirectional. Therefore, the direction of the SSC region was a focus of comparative genome analysis. The genome assembly of Baekdansim consisted of a single contig and was used as a resource to investigate the direction of the SSC region. In a previous study, the primary hypothesis was that the direction of the SSC may have been due to a recombination event between the two IR regions. The alternative hypothesis was that the direction of the SSC region depended on the assembly method; the precise direction of the SSC region was unknown because a short-read-based sequencing platform was used (Cheng et al., 2020). This study showed that the whole chloroplast sequence, which was obtained as a single contig, spanned the whole LSC-IR-SSC area and that the gene order of close species of the family Malvaceae with an inverted SSC structure was exactly reversed. Based on these results, it could be concluded that the SSC direction was changed because of a misassembly induced by constraints of the short read-based sequencing platform. To perform an accurate comparative analysis of species of the tribe Hibisceae, the mis-assembled section was corrected based on the SSC strand derived from long-read sequencing using an in-house Python script.

### 3.2 Genome structure and gene content

The complete chloroplast genome of Baekdansim contains 113 genes, including 79 protein-coding genes, 4 rRNAs, and 30 tRNAs. Multiple genes were duplicated in the IR regions, including 5 protein-coding genes (*rpl2, rps7, rpl23, ndhB*, and *ycf2*), 7 tRNAs (*trnA-UGC, trnI-GAU, trnN-GUU, trnV-GAC, trnL-CAA, trnR-ACG*, and *trnI-CAU*), and 4 rRNAs (*rrn5, rrn4.5, rrn23*, and *rrn16*). As generally observed in other angiosperms, 18 intron-containing genes were also detected in the Baekdansim chloroplast genome (Redwan et al., 2015; Mo et al., 2020). Eighteen genes contained one or more introns; of which, 11 encoded proteins (*atpF, clpP1, ndhA, ndhB, pafI, petB, petD, rpoC1, rps16, rpl2*, and *rpl16*), 6 encoded tRNAs *(trnA-UGC, trnG-UCC, trnI-GAU, trnK-UUU, trnL-UAA*, and *trnV-UAC*), and 1 encoded an rRNA (*rrn23*). The *rps12* gene exhibited a trans-spliced form with its 5′ terminal present in the LSC region, and its 3′ end had a single copy present in each of the two IR regions, similar to the patterns observed in other terrestrial plants (Hildebrand et al., 1988; Lee et al., 2019) (**Supplementary Table 2**). A single copy of *ycf1* was present due to its position in the SSC region instead of in the IR regions. This finding is consistent with a previous study of *Distemonanthus benthamianus*, in which IR contraction was observed (Bai et al., 2021). According to previous reports, the plastome length varies according to the IR length, suggesting that the chloroplast length of *H. syriacus* is also affected by this IR length variation (Zhu et al., 2016; Liang et al., 2022; Lu et al., 2022).

### 3.3 Repeat analysis

Repeat motifs, which are widely distributed in chloroplast genomes, play an important role in genome evolution (Powell et al., 1995; Yang et al., 2011; Xue et al., 2012; Liu et al., 2018). The number of SSR motifs in the Baekdansim plastome was investigated using MISA software. We identified 109 SSRs (microsatellites) among which 81 (74.3%) consisted exclusively of A/T. Similar to a previous report, the majority of mononucleotide repeats were A/T, and most SSRs consisted of mononucleotide repeats (George et al., 2015). We found 82 (75.2%) mono-, 10 (9.3%) di-, 7 (6.4%) tri-, 6 (5.5%) tetra-, and 4 (3.7%) penta-nucleotides (**Supplementary Table 3**).

Long-repeat elements are crucial for not only structural variation in chloroplast genomes but also intermolecular recombination, leading to genome diversity (Park et al., 2017; Kong et al., 2021). Complex repeats in Baekdansim were discovered using the REPuter program. The repeat length ranged from 30 bp to 78 bp, which corresponds to the typical range of other plastomes (Greiner et al., 2008; Li et al., 2017). The most abundant repeats were forward repeats, followed by palindromic repeats and reverse repeats (**Supplementary Figure 1**). These identified repeats and SSRs will be useful for developing molecular markers for genetic diversity and evolution studies.

### 3.4 Comparison of chloroplast genome structure and nucleotide diversity

**Figure 2:**
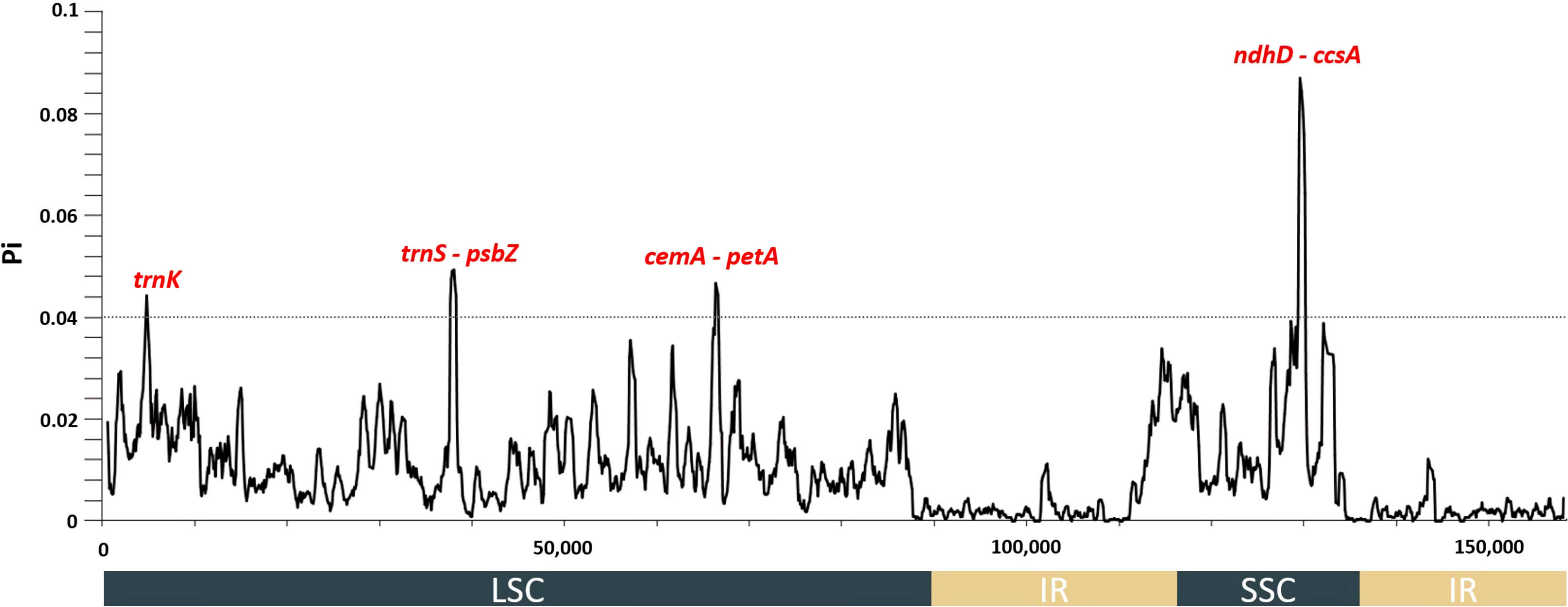
Nucleotide diversity values among 13 species of the tribe Hibisceae were calculated using whole plastomes. Mutational hotspots (Pi > 0.04) are denoted above the corresponding gene position.

Only *Abelmoschus* species contained *rps3b, rps19b*, and *rpl22b*, but other genes were detected in all 13 species of the tribe Hibisceae (**Supplementary Figure 2**). IRs were more conserved than LSC and SSC sections, while non-coding regions were more divergent than coding regions (**Supplementary Figure 3**). These results were congruent with findings in other land plant species (Jian et al., 2018; Kim et al., 2020). In addition, the intergenic spacer regions among several gene pairs varied remarkably in chloroplast genomes of the tribe Hibisceae. For instance, these regions differed markedly among *trnH-GUG-psbA*, *trnK-UUU-rps16*, *trnF-GAA-ndhJ*, *atpB-rbcL*, *rps12-trnV-GAC*, *ndhl-ndhG*, and *ndhD-ccsA.* The highest level of nucleotide diversity was identified in a few of these intergenic spacer regions. Collectively, these results suggested these regions might indicate the rapid evolution of the tribe Hibisceae (**Figure 2**). The nucleotide diversity among the 13 chloroplast genomes of the tribe Hibisceae was calculated. The results indicated four highly divergent hotspots, *trnK, trnS-psbZ, cemA-petA*, and *ndhD-ccsA*, with a threshold of 0.04. All of these hotspots were found in single-copy (LSC and SSC) regions. The most variation was observed in the *ndhD-ccsA* region (0.08703) (**Figure 2**). It will be important to determine whether these regions could be employed as DNA barcodes to clarify close relationships within the tribe Hibisceae.

**Figure 3:**
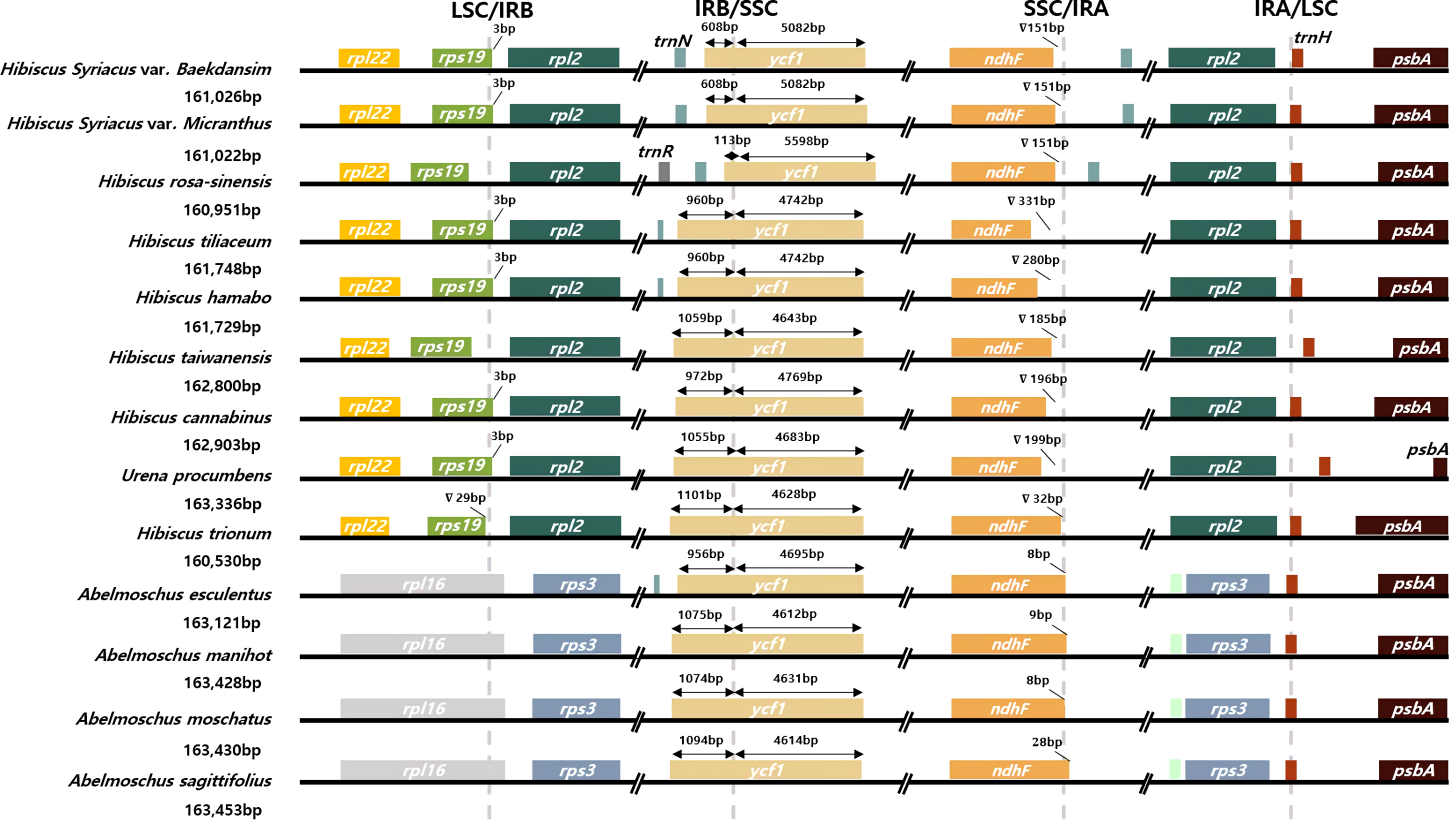
Comparison of the junction sites among 13 species of the tribe Hibisceae.

Contraction or expansion of the single-copy and IR regions commonly occurs in various angiosperms (Jian et al., 2018; Li and Zheng, 2018; Henriquez et al., 2020; Guo et al., 2021; Liu et al., 2021). These alterations are considered a major mechanism that causes size variation of the chloroplast genome and evolutionary events (Zhu et al., 2016; Liang et al., 2022; Lu et al., 2022). Four junctions between the two single-copy regions and the two IR regions of 12 representative species of the tribe Hibisceae were thoroughly compared to examine chloroplast genome variation in the tribe Hibisceae (**Figure 3**; **Supplementary Table 4**). IR regions are relatively conserved in the *Hibiscus* genus; nonetheless, considerable contraction and expansion occur in the IR/SSC regions. The *ycf1* gene was displaced from the IRB to the SSC region at the IRB/SSC boundary in the chloroplast genomes of *H. syriacus* and *H. rosa-sinensis* by 608 bp and 113 bp, respectively. This movement indicates IR contraction in the chloroplast genomes of these species. The *ndhF* gene was shifted from the SSC region to the IRA region, and *rpl16* was shifted from the LSC region to the IRB region in *Abelmoschus* species, according to comparisons between *Hibiscus* and *Abelmoschus* species. The longer chloroplast genome in *Abelmoschus* than in the *Hibiscus* species could be attributed to this IR expansion. In previous reports, shifting of genes to the IR regions or SSC region led to size variation of IR regions in the family Malvaceae. The current study showed that the overall length of the plastome was affected by this size variation shown in previous reports (Dugas et al., 2015; Wang et al., 2016; Guo et al., 2021).

### 3.5 Putative RNA-editing sites

RNA editing is a post-transcriptional regulation mechanism that can result in the alteration of ribonucleotides at specific sites (Maier et al., 1996). According to previous research, C-to-U conversion is the primary factor responsible for RNA editing (Tsudzuki et al., 2001). Two possible RNA-editing sites were predicted in the start codon of *ndhD* and the stop codon of *rpoC1* in the chloroplast genomes of the tribe Hibisceae (**Supplementary Figure 4**). In particular, *ndhD* was edited at a high level in *Galium* species. In addition, the start codon (ACG) of *ndhD* in nine species of the family Rubiaceae was affected by an RNA-editing event, which is consistent with the pattern of RNA editing in species of the tribe Hibisceae (Zhang et al., 2019). RNA editing regulates gene expression and has a substantial impact on translation (Maier et al., 1996). RNA editing in the protein-coding region results in codon alterations that lead to amino acid substitution, which may affect the stability of the tertiary structure of proteins (Gommans et al., 2009). Furthermore, these alterations have been related to the generation of genetic diversity, which is a factor in adaptive evolution (Gommans et al., 2009). In tobacco, frequent editing occurs in *ndh*, which encodes the subunits of a plastid NAD(P)H dehydrogenase (Hirose and Sugiura, 1997; Fiebig et al., 2004). Expression of *ndhD* in the tobacco chloroplast, as determined by RNA editing, to create the start codon was greatest in young and photosynthetically active leaves (Hirose et al., 1997). In addition, although *ndh* gene products are dispensable under normal growth conditions, editing is likely essential for the appropriate function of the Ndh protein complex and cyclic electron flow under stress conditions. Fixation of a mutation in a non-essential gene allows plasticity and sufficient time for the evolution of a mutation-compensating editing capacity under moderate selective pressure (Fiebig et al., 2004). Therefore, the occurrence of RNA editing in *ndhD* at the same site in all species of the tribe Hibisceae could be regarded as a result of environmental adaptation. In general, species of the tribe Hibisceae are tolerant of abiotic stresses such as cold, drought, and salt stresses (Zhan et al., 2019; An et al., 2020; Eo et al., 2020; Mahougnon et al., 2021; Chen et al., 2022). Thus, the fixation of RNA editing might have occurred *via* long periods of adaptation to environmental changes during evolution.

### 3.6 Codon usage pattern and phylogenetic analysis

**Figure 4:**
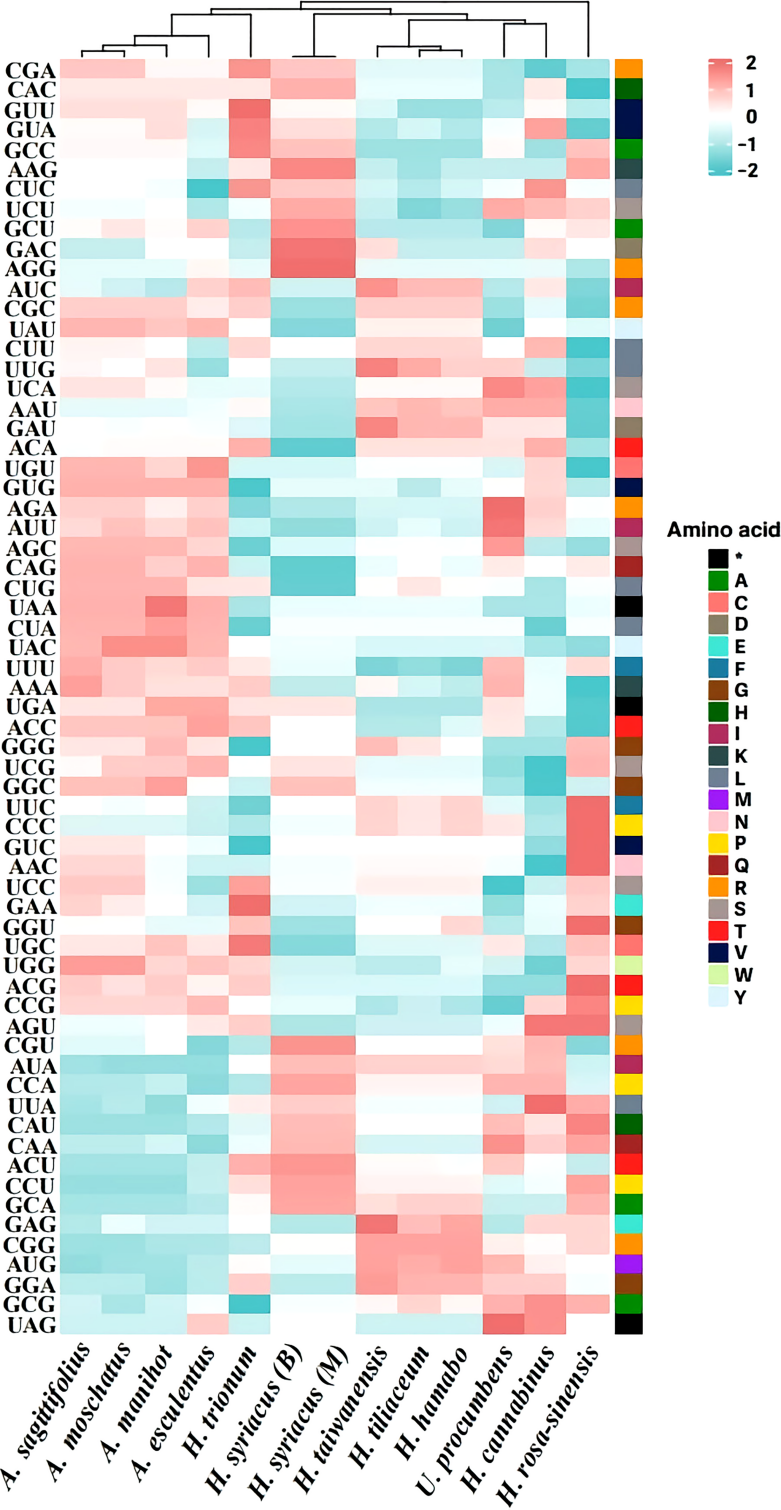
Relative synonymous codon usage (RSCU) pattern of chloroplast genes among 13 species of the tribe Hibisceae.

Many terrestrial plants exhibit codon usage bias, which is considered to play a substantial role in regulating translation dynamics (Du et al., 2020). Recent studies have demonstrated that codon preferences significantly influence the evolution of the chloroplast genome by balancing natural selection and mutational biases (Akashi and Eyre-Walker, 1998; Raubeson et al., 2007; Hershberg and Petrov, 2008). In this study, the RSCU of protein-coding genes in the chloroplast genome of the tribe Hibisceae was investigated and identified (**Figure 4**). Among the protein-coding codons, the most frequently encoded was leucine, followed by those encoding arginine and alanine; the GAC codon, which encodes aspartic acid, had the lowest usage frequency. If neutral mutations occur at the third codon position, GC and AT would equally present among the codon groups within a chloroplast genome (Zhang et al., 2007). However, most codons showed a bias toward an A/U ending, and these findings are consistent with those observed in other chloroplast genomes (Yan et al., 2019; Du et al., 2020). Previous studies revealed that this unequal usage of nucleotides derived from mutation selection and natural selection was the primary driver of codon bias in angiosperms (Nie et al., 2014; Wang et al., 2020; Zhang et al., 2021). These findings indicate that the high proportion of A/U-ending codons in the chloroplast genome, along with the selective pressure of the chloroplast genome of the tribe Hibisceae, may have driven several degenerate codon biases.

**Figure 5:**
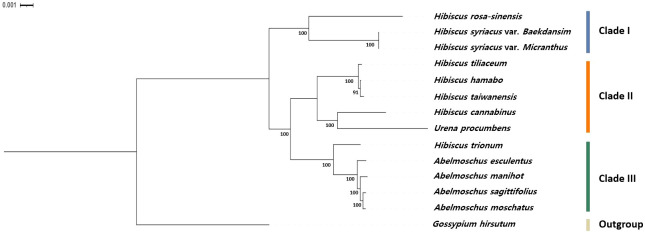
Phylogenetic relationships based on the whole chloroplast genomes of 13 species of the tribe Hibisceae. The bootstrap values were based on 1000 replicates and are denoted next to the branches.

Moreover, species within the tribe Hibisceae were largely clustered into five groups by the RSCU pattern (Group I: *H. syriacus*, Group II: *H. taiwanensis*, *H. tiliaceum*, and *H. hamabo*, Group III: *Urena procumbens* and *H. cannabinus*, Group IV: *H. rosa-sinensis*, Group V: *Abelmoschus sagittifolius*, *A. moschatus*, *A. manihot*, *A. esculentus*, and *H. trionum*) (**Figure 4**). Phylogenetic analysis was performed on an alignment of the whole chloroplast genome sequences of 13 species of the tribe Hibisceae (**Figure 5**). It is noteworthy that *H. trionum* formed a clade with *Abelmoschus* species. This tendency was linked to the codon usage pattern of protein-coding genes in the tribe Hibisceae (**Figure 4**). Despite belonging to a distinct genus, it was assumed that the similarity of codons with other genera may have affected clade formation among other genera. The association between codon usage patterns and the phylogenic topology inferred from the whole chloroplast genome provides strong support for the hypothesis that nucleotide bias induces codon bias.

## 4 Conclusion

In this study, the complete chloroplast genome of Baekdansim was constructed *via* a long-read sequencing platform for the first time. Through comparisons among species of the tribe Hibisceae, we found that four mutational hot spots could be used to develop DNA barcodes. Furthermore, we identified fixation of candidate RNA-editing sites, a preference for A/U-terminated codons, and a notable codon usage pattern related to phylogenetic relationships. Comparison analysis of whole chloroplast genomes of the tribe Hibisceae offers a valuable genomic resource for understanding the evolution and adaptation of this tribe and its relatives.

## Data availability statement

The datasets presented in this study can be found in online repositories. The names of the repository/repositories and accession number(s) can be found in the article/**Supplementary Material**.

## Author contributions

HK and Y-MK conceived and designed this study; HK, A-YS, and SH analyzed the data; HK and Y-MK wrote the manuscript; HK, A-YS, and Y-MK revised the manuscript; Y-MK supervised this study. All authors contributed to the article and approved the submitted version.
