# Supplementary material for: The complete chloroplast genome of Hibiscus syriacus using long-read sequencing: Comparative analysis to examine the evolution of the tribe Hibisceae
Source: Front Plant Sci. 2023 Feb 2;14:1111968. doi: 10.3389/fpls.2023.1111968 (PMC9931742; doi:10.3389/fpls.2023.1111968)
Supplement: Supplementary file 1 [file DataSheet_1.docx]

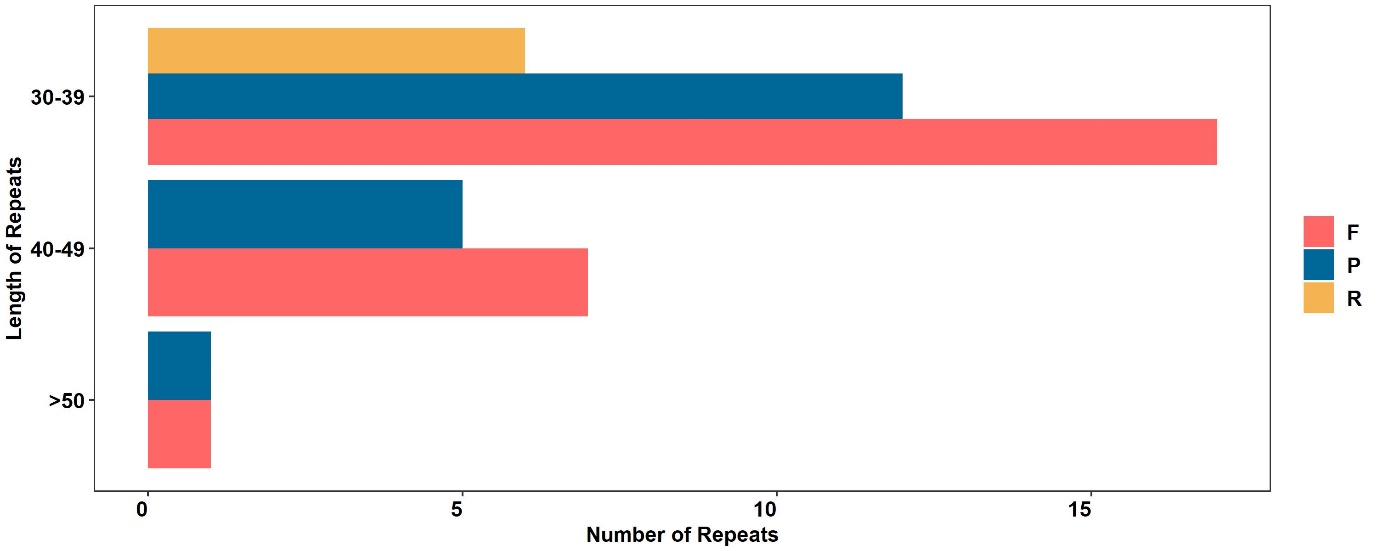


**Supplementary Figure 1.** Length and frequency of repeats in *H. syriacus* var. *Baekdansim* chloroplast genome.


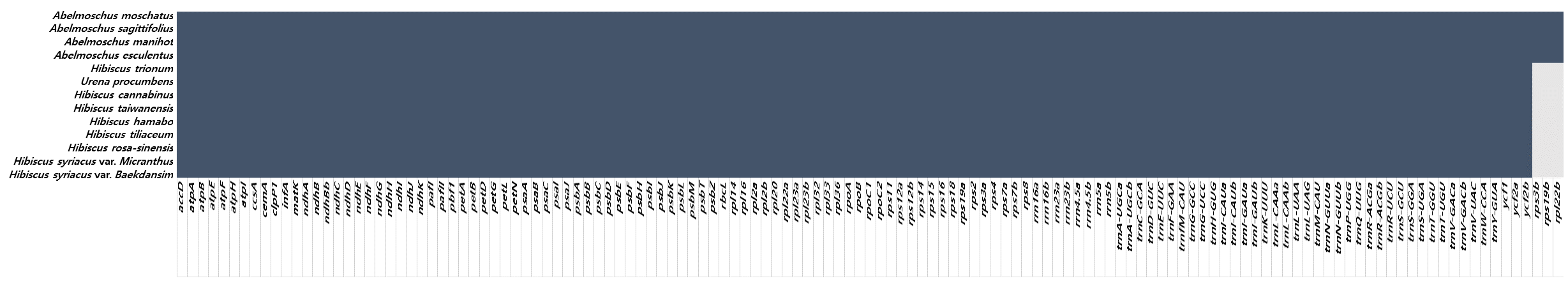


**Supplementary Figure 2.** Presence and absence of genes in 13 species belonging to the tribe Hibisceae.


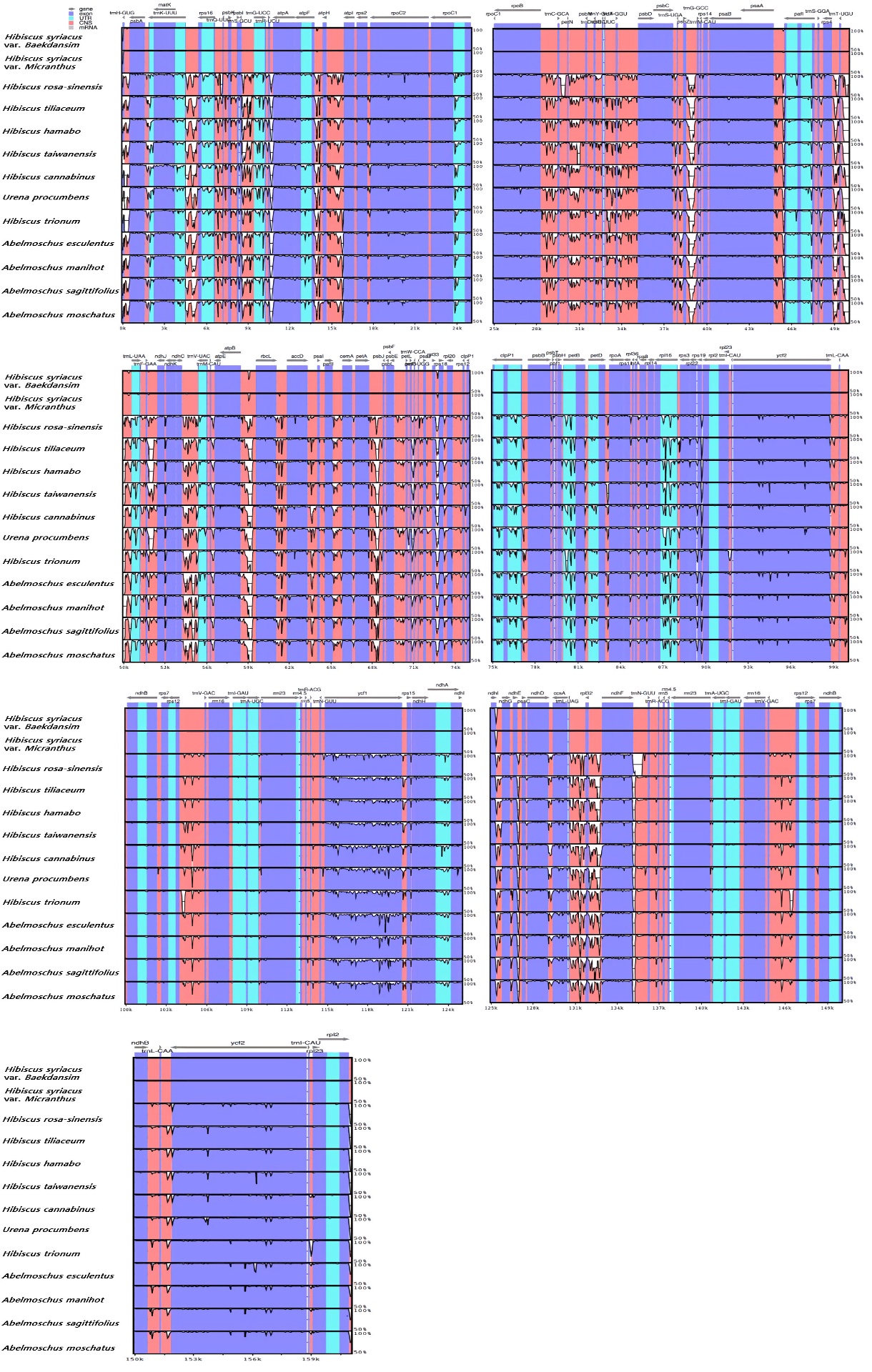


**Supplementary Figure 3.** Comparison of 13 chloroplast genomes using mVISTA program. Genome regions are color-coded as protein-coding (exon) and conserved noncoding sequences (CNS).

**Supplementary Figure 4.** Putative RNA-editing sites in the start and stop codon
